# Behavioral Immune System and Ingroup Derogation: The Effects of Infectious Diseases on Ingroup Derogation Attitudes

**DOI:** 10.1371/journal.pone.0122794

**Published:** 2015-03-27

**Authors:** Qi Wu, Chuan Tan, Bo Wang, Ping Zhou

**Affiliations:** 1 Cognition and Human Behavior Key Laboratory of Hunan Province, Department of Psychology, Hunan Normal University, Changsha, China; 2 Department of Psychology, School of Social Development, Central University of Finance and Economics, Beijing, China; The University of New South Wales, AUSTRALIA

## Abstract

From evolutionary reasoning, we derived a novel hypothesis that ingroup derogation is an evolved response of behavioral immune system which follows the smoke detector principle and the functional flexibility principle. This hypothesis was tested and supported across three experiments. In Experiment 1, participants’ group membership was manipulated by using a minimal group paradigm. The results indicated that mere social categorization alone — a heuristic cue that implies the differentiation between "us" and "them" — was sufficient to elicit ingroup derogation among Chinese participants, and, such an intergroup bias was positively associated with the perceived vulnerability to diseases, which was also more consistently associated with ingroup attitudes. Experiment 2 extended and partially replicated Experiment 1 by showing that when there were cues of diseases in the immediate physical environment, Chinese participants exaggerated their attitudes of ingroup derogation. The results also showed that this effect was mainly driven by outgroup attraction. Experiment 3 changed the method of disease manipulation, and found that Chinese participants responded more strongly to disease cues originating from ingroup members and that they endorsed more ingroup derogation attitudes even when the ingroup and outgroup members were both displaying cues of diseases. Taken together, these results reveal the previously unexplored effects of infectious diseases on ingroup derogation attitudes, and suggest an interesting linkage between the evolved behavioral immune system and the ingroup derogation.

## Introduction

For a very long time, parasitic organisms (e.g. bacteria, viruses, parasites) have posed great threats to the reproductive fitness of many species. Consequently, the organisms have evolved two different immune systems to defend against these pathogens [1, 2]. The physiological immune system detects and mobilizes physiological responses to eliminate the pathogens entering the body. The behavioral immune system functions as the first level of defense against pathogens. It detects the presence of pathogens in the environment and facilitates the avoidance of those pathogens before they enter the body, thus keeping the organism from actually mounting to a costly immune response. Researchers have observed this behavioral immune response across many animal species [3–5]. In recent years, the behavioral immune system has received much attention in the study of human behavior [1, 2]. A burgeoning body of literature has documented the effects of perceived threat of pathogens on human personality [6, 7], emotion [8], attention [9], perception [10], memory [10], sexual behavior [7, 11, 12], social influence [13–19], and so on.

The behavioral immune system is also involved in the evolution of a ubiquitous tendency of ingroup favoritism (also known as ingroup bias) [10, 13, 20–34]. Since the organism mainly evolves resistance to local parasitic organisms, rather than to those evolving in nearby regions [31–33], outgroup members often carry novel pathogens infectious to ingroup members. Under conditions of high pathogen prevalence, a psychological mechanism facilitating the association with ingroup members and avoidance of outgroup members should be favored by natural selection [31–36].

However, a similar but completely opposite phenomenon of ingroup favoritism has also been reported in the study of intergroup bias, a phenomenon called ingroup derogation [37–48]. Results showed that, when participants were from the inferior social groups [37–39], or from East Asian cultures [40–44], or when the targets were deviant ingroup members [45–48], participants showed a preference to and affinity for outgroup members instead of ingroup members. This strange anti-us behavioral tendency was particularly prevalent in East Asian cultures [40–44, 49–54]. For example, researchers have even found that Mainland Chinese rate the faces and names of outgroup members as more beautiful and better than their ingroup counterparts [44].

Currently, researchers cannot well explain ingroup derogation in terms of proximate cause [43, 44], nor can they explain it within the evolutionary framework: Natural selection should have favored the individuals who preferred their ingroups, and the individuals who preferred outgroups would be selected against by natural selection [31–36]. Therefore, from an evolutionary perspective, preference for outgroup members is not considered as adaptations but be considered as maladapations, which makes it very difficult to explain the prevalence and persistence of ingroup derogation.

One important assumption underlying the evolutionary theory of ingroup favoritism is that the pathogen threat posed by outgroup members should be much greater than the pathogen threat posed by ingroup members, otherwise it would be more adaptive to avoid ingroup members rather than avoiding outgroup members. However, this assumption may be problematic, considering sometimes the pathogen threat incurred by ingroup interactions actually could be greater than the pathogen threat posed by outgroup members: Interactions between ingroup members are much more frequent than interactions between an individual and the outgroup members [36], which creates more chance of spreading an infectious disease via an ingroup member; if there are outbreaks of some recent emerging dangerous epidemics to which ingroup members are still not immune, or the pathogen load within the local habitat of ingroup members become much higher than the pathogen load within the local habitat of outgroup members, it would be much more easier to catch an infectious disease via an ingroup member than via an outgroup member. Under such circumstances, it would be more adaptive to derogate, to dislike, to feel disgusting toward, and to avoid ingroup members than to bond with them. A favoritism toward outgroup members would help our ancestors to abandon their original groups and to associate with other groups in order to find more favorable habitats. If such situations did occur recurrently in the long stretch of evolutionary history of human species, then our ancestors probably would have evolved psychological mechanisms to facilitate a behavioral response of favoring outgroup members over ingroup members under particular ecological conditions.

Some empirical evidence has implied that this hypothesis could be supported. For example, China has been found to have higher pathogen prevalence than Europe by both historical and contemporary measures of pathogen prevalence [13], and China also happens to be the area where participants endorse ingroup derogation attitudes [43, 44]. Contrary to previous findings on the positive relationship between behavioral immune system and social conservatism (for meta-analysis, see [18]), recent studies have also shown that there were no associations or inconsistent associations between local pathogen prevalence and ingroup favoritism [19, 55, 56]. Studies have also showed that the relationship between parasite stress and strength of family ties is better to be described by a quadratic function (i.e., the strength of family ties drops when the parasite stress give rises to a certain level) than by a simple linear model [57], and that, in some areas (e.g., Africa), the correlations between parasite stress and ingroup assortativeness were reported to be negative rather than positive [31]. Some researchers have even proposed that, in face of extremely high parasite stress, ingroup investment may not be optimal and should be reduced because extreme parasite stress yields extrinsic mortality [58]. Regretfully, these empirical and theoretical evidence can only support our hypothesis indirectly. More direct empirical evidence is needed for a solid conclusion.

### The Current Study

The behavioral immune system follows the smoke detector principle and the functional flexibility principle [1, 2, 36]. The smoke detector principle indicates the behavioral immune system is prone to make false-positive errors. It responds to heuristic cues which imply the presence of diseases. The functional flexibility principle dictates that, under circumstances in which individuals are easy to be infected or merely perceive themselves to be vulnerable to pathogen infection, the activation of behavioral immune system is stronger. According to our hypothesis, ingroup derogation is an evolved response of the behavioral immune system. Such a mechanism should also follow the smoke detector principle and the functional flexibility principle. Then, ingroup derogation should not only exist in actual social groups [37–48]. Mere social categorization alone—a heuristic cue that implies the differentiation between "us" and "them" [59, 60]—should be sufficient to produce this bias (i.e., smoke detector principle), which should be particularly stronger when the individuals feel vulnerable to diseases, when there are cues of diseases in the immediate physical environment, or when there are people who display disease cues in the immediate social environment (i.e., functional flexibility principle). Furthermore, such a mechanism should respond more consistently to ingroup members and respond more strongly to the disease threat incurred by ingroup members because it was designed to deal with a special ecological condition in which the greater disease threat was brought by the ingroups instead of the outgroups. In the present study, we tested our hypothesis by three experiments. We mainly focused on the ingroup derogation among mainland Chinese.

The minimal group paradigm categorizes people into artificially distinct groups on the basis of arbitrary criteria, such as whether they have a "red" personality type or a "green" personality type based on a bogus personality test [59], which provides group-categorization heuristics to one's actual social group membership [60]. In Experiment 1, by using the minimal group paradigm, we tested whether Chinese participants would show ingroup derogation when the cues denoting one's group were only artificial labels heuristically associated with one's actual social group membership. We further examined whether such an intergroup bias was positively associated with individual differences in perceived vulnerability to diseases in Experiment 1. Considering that ingroup derogation is assumed to be an adaptation to a special situation in which ingroup members pose more threat of diseases than outgroup members, we predicted that when facing both ingroup and outgroup members, the behavioral immune system would mainly function to avoid ingroup members for Chinese participants. Thus, in Experiment 1, we also expected to find a stronger negative association between perceived vulnerability to diseases and ingroup attitudes than outgroup attitudes.

In Experiment 2, we extended and partially replicated Experiment 1 by using experimental methods to test whether the presence of external disease cues in the immediate physical environment would lead Chinese participants to exaggerate ingroup derogation attitudes.

According to our hypothesis, ingroup derogation is a special adaptation designed to manage the disease threat incurred by social interactions (especially to ingroup members). Such a mechanism should not only respond to cues of diseases from the immediate physical environment. It should also be more activated when there are people who display disease cues in the immediate social environment. We predicted that, when facing the ingroup and outgroup members who may or may not have infectious diseases, for the Chinese participants, the main function of behavioral immune system would be to avoid the ingroup members displaying the cues of diseases. We expected the effects of disease cues to be stronger for ingroup members than for outgroup members and that the degree of ingroup derogation attitudes to be exaggerated even when ingroup and outgroup members are both displaying cues of diseases. These possibilities were tested in Experiment 3.

In all experiments, we used the degree of acceptance (i.e., acceptance of a specific group member as a partner to work with) as the measure of participants' preference for a specific group membership (see [26] for a similar measure of intergroup bias). Previous studies of ingroup favoritism have shown that participants incline to affiliate to and cooperate with their ingroup members rather than with outgroup members [25, 26, 31–33, 35, 36, 61–63].

## Experiment 1

In Experiment 1, we tested whether Chinese participants would show ingroup derogation when being provided with heuristic cues of one's actual social group membership and whether such an intergroup bias was positively associated with individual differences in perceived vulnerability to diseases. We also examined the relationships between perceived vulnerability to diseases and ingroup/outgroup attitudes.

### Ethics Statement

The experimental procedures were approved by the IRB of the Institute of Psychology, Hunan Normal University. All participants and the caretakers of the participants aged under 18 years provided written informed consent before taking part in the experiment and were debriefed after the experiment. The individual in this manuscript has given written informed consent (as outlined in PLOS consent form) to publish these case details.

### Participants and Design

Sixty Chinese undergraduate or postgraduate students (30 males and 30 females), aged 17–30 years (mean age: 23.2 years, *SD* = 2.73), participated in this experiment for monetary compensation. A 2 (personality type: red, green) × 2 (category label: ingroup, outgroup) mixed-model experimental design was used, with personality type being the between-subjects factor and category label being the within-subjects factor.

### Materials and Procedure

A bogus personality test was used to create the artificial groups. Forty questions from the Eyesenck Personality Questionnaire (EPQ) [64] were presented to participants one at a time on a computer screen. Each question remained on the screen until a response was made. Participants were instructed to press a button to indicate whether or not they fit the presented description. After they completed the test, the computer ostensibly analyzed their responses, and informed them that they were either a "red" or a "green" personality type.

The individual differences in perceived vulnerability to diseases were measured by The Perceived Vulnerability to Disease scale (PVD) [65], a 15-item measure used in several previous studies [10, 12, 14, 20, 26, 27, 66] to assess the perceived personal susceptibility to the transmission of diseases. The scale included two subscales that respectively measure perceived infectability (α = 0.55) and germ wariness (α = 0.68). We also used PVD as a single scale (α = 0.76). Higher scores on these measures indicate greater perceived vulnerability to diseases.

**Fig 1:**
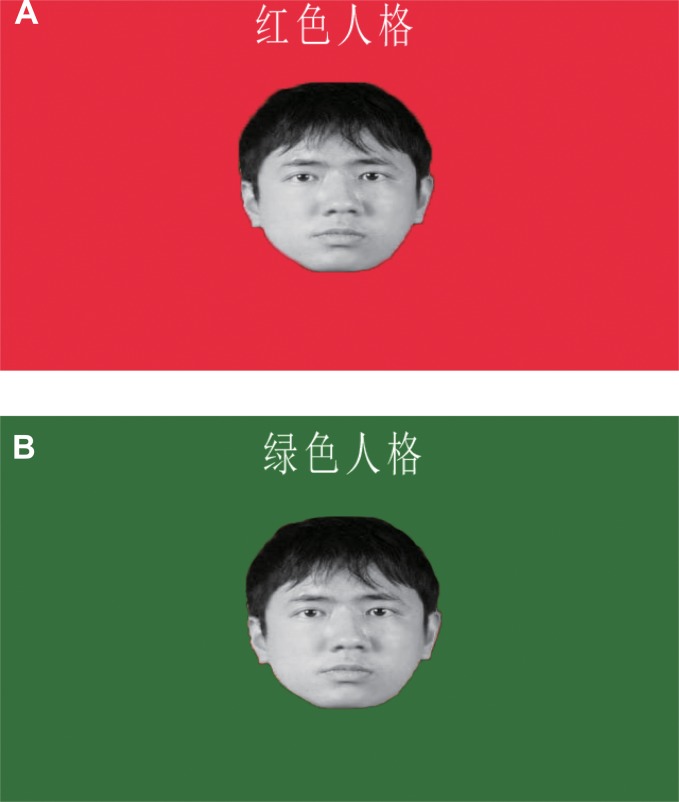
Example of stimuli in Experiment 1. The facial stimuli that was labeled as red personality type is shown in A, while the facial stimuli that was labeled as green personality type is shown in B. The facial stimuli was presented at the center of screen. The label of red personality type (A) or green personality type (B) was inscribed at the top of screen.

Eighty gray-scale facial images of Chinese college-age males and females with neutral facial expressions were chosen as the stimuli [67–69]. These images had already been employed in our previous study [44]. They consisted of two image sets matched on the degree of beauty [44] and they were completely novel to all participants. Twenty college students who did not participate in the formal experiments rated the degree of acceptance for these two sets. The participants had to indicate "to what extent would you want to work together with the person shown on the screen in the next experiment" on an 8-point scale (1 = "definitely not" to 8 = "definitely like to"). A pairwise *t*-test showed that there was no difference for the degree of acceptance between the first set (*M* = 4.29, *SD* = 0.18) and the second set (*M* = 4.38, *SD* = 0.26), *t*(19) = -1.76, *p* > 0.05. As for the thirty participants assigned to red personality type, fifteen of them were exposed to a stimuli sequence in which the first facial image set was labeled with red personality type and the second image set was labeled with green personality type. The other fifteen were exposed to a stimuli sequence in which the second facial image set was labeled with red personality type, whereas the first image set was labeled with the green personality type. The same procedure was employed for the thirty participants assigned to green personality type. Thus each image set has equal probability of being labeled as ingroup or outgroup members. Each face was presented at the center of the screen, with a background color that was identical to its personality type (red and green were the background colors for red and green personality types, respectively). The label of personality type was placed at the top of the background in order to label the face (as shown in Fig. 1).

After providing informed consent, participants were instructed that they would take a computerized personality test at first. They were told that the red personality type was not necessarily better than the green personality type or vice versa, and they were informed that the experiment was designed to investigate psychological differences between these two different personality types. Given no further description of the personality types, they were then given a green or red identity tag to wear, and told it was to identify their particular personality type (see [59] for a similar procedure). Participants were then instructed that they would view faces on the computer screen, and that the background color and the label displayed on the top of the screen would denote whether that person had a red personality type or a green personality type. They were instructed that their task was to rate "to what extent would you want to work together with the person shown on the screen in the next experiment" on an 8-point scale (1 = "definitely not" to 8 = "definitely like to") for these faces. The faces were presented one at a time and each face remained on the screen until the response was made. Faces were randomly presented for each participant. After completing this face appraisal task, participants were instructed to complete the PVD scale.

### Results

**Fig 2:**
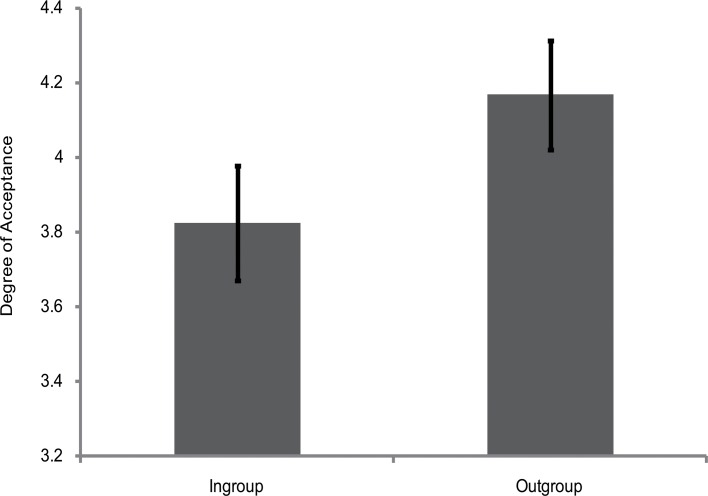
Degree of acceptance of faces labeled as ingroup members and outgroup members in Experiment 1. Error bars represent standard errors.

The rating scores of the face appraisal task were subjected to a 2 (personality type: red, green) × 2 (category label: ingroup, outgroup) mixed-model analysis of variance (ANOVA). The main effect of category label was significant [*F*(1, 58) = 4.44, *p* < 0.05, η_*p*_^2^ = 0.07] (see Fig. 2), indicating that participants were more inclined to affiliate with outgroup members than with ingroup members. However, the main effect of personality type [*F*(1, 58) = 0.32, *p* > 0.05, η_*p*_^2^ = 0.006] and the interaction between category label and personality type [*F*(1, 58) = 0.01, *p* > 0.05, η_*p*_^2^ <. 001] were not significant. Therefore, participants showed ingroup derogation when the cues denoting one's group were only heuristically associated with one's actual social group membership.

**Fig 3:**
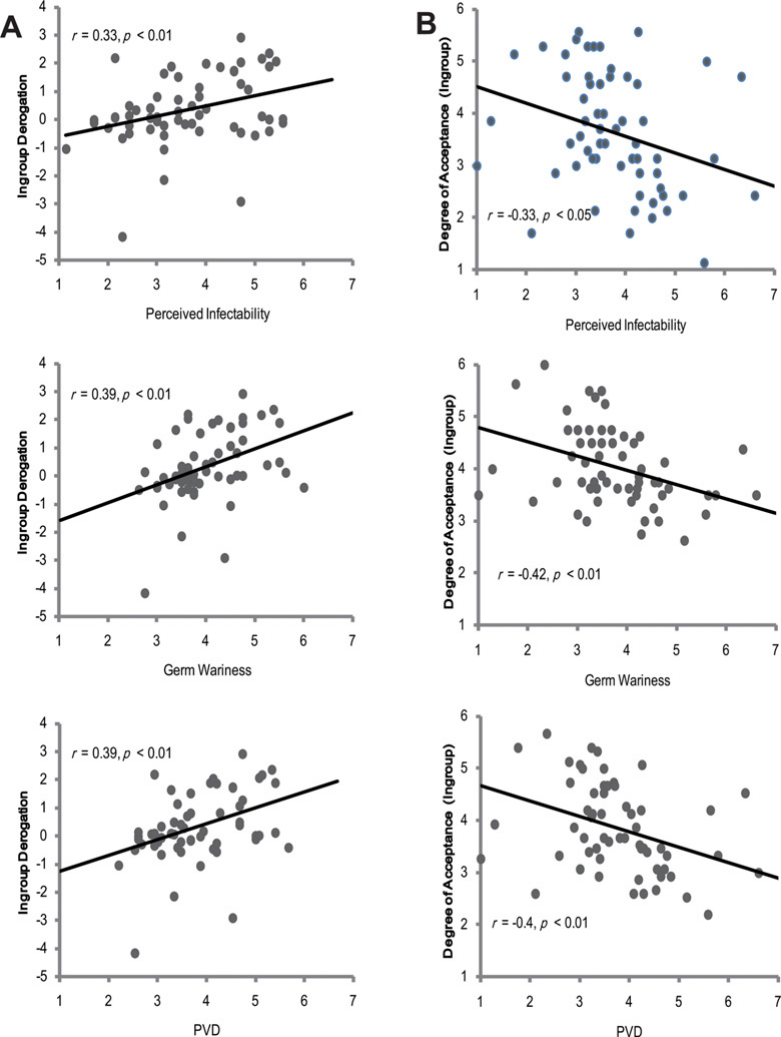
Correlations between the PVD measures and the scores of ingroup derogation and ingroup attitudes.

The rating scores of outgroup members in the face appraisal task were then subtracted by that scores of ingroup members to create a composite score of ingroup derogation. A positive score reflects ingroup derogation, whereas a negative score reflects ingroup favoritism. To assess the relationship between ingroup derogation (*M* = 0.34, *SD* = 1.25) and perceived vulnerability to diseases, correlational analyses were carried out. The Pearson product-moment correlations between ingroup derogation and the PVD measures (see Fig. 3A) were as follows: (a) perceived infectability: *r* = 0.33, *p* < 0.01; (b) germ wariness: *r* = 0.39, *p* < 0.01; (c) PVD: *r* = 0.39, *p* < 0.01. These results indicated that there was positive association between the perceived vulnerability to diseases and the degree of ingroup derogation.

Further analyses revealed that such positive correlation was mainly driven by a negative correlation between ingroup attitudes and perceived vulnerability to diseases (see Fig. 3B). Rating scores of ingroup members and PVD measures were negatively correlated: (a) perceived infectability: *r* = -0.33, *p* < 0.05; (b) germ wariness: *r* = -0.42, *p* < 0.01; (c) PVD: *r* = -0.4, *p* < 0.01. No significant correlations were found between rating scores of outgroup members and PVD measures: (a) perceived infectability: *r* = 0.02, *p* > 0.05; (b) germ wariness: *r* = -0.003, *p* > 0.05; (c) PVD: *r* = 0.01, *p* > 0.05. These results were consistent with the prediction that the perceived vulnerability to diseases would be more strongly correlated with ingroup attitudes than with outgroup attitudes.

## Experiment 2

In Experiment 2, we tested whether the presence of external disease cues in the immediate physical environment would lead Chinese participants to exaggerate their attitudes of ingroup derogation by setting up different experimental environments. Similar experimental procedures have been employed in the study of evolution of ingroup favoritism: Participants were instructed to clean the keyboard and their hands to provide them a cue of protection against infectious diseases [20]. We adopt this logic and applied it to our experiment. To provide the participants with external disease cues, participants in the disease condition had to finish this experiment by using a very dirty keyboard (with dust, mud, glue, and some paintings on its surface), while the participants assigned to the control condition had to finish the experiment by using a normal keyboard.

To test whether the environmental setting of the disease condition could effectively provide a cue of disease, we conducted a pilot study (*n* = 40) in which participants in the disease condition and the control condition were respectively instructed to type on the dirty keyboard and the normal keyboard for 10 minutes (approximately the length of time that the formal experiment would take) and then they were instructed to rate the "possibility of getting a disease by using this keyboard" on a 9-point scale (1 = "very easy", 9 = "very hard"). An independent *t* test showed significantly lower ratings in the disease condition (*M* = 4.20, *SD* = 1.15) than in the control condition (*M* = 7.00, *SD* = 1.17), *t*(38) = -7.63, *p* < 0.01. Thus, the pilot study provided the evidence that the manipulation of environmental setting was effective.

### Ethics Statement

The experimental procedures were approved by the IRB of the Institute of Psychology, Hunan Normal University. All participants provided written informed consent before taking part in the experiment and were debriefed after the experiment.

### Participants and Design

Eighty Chinese undergraduate or postgraduate students (40 males and 40 females) aged 19–26 years (mean age: 22.39 years, *SD* = 2.04) were paid to participate in this experiment. A 2 (personality type: red, green) × 2 (category label: ingroup, outgroup) × 2 (environmental setting: disease condition, control condition) mixed-model experimental design was used, with personality type and environmental setting being the between-subjects factors while category label being the within-subjects factor.

### Materials and Procedure

The bogus personality test which was used to create artificial groups and the facial stimuli that were employed by this experiment were identical to those of Experiment 1.

The procedure of Experiment 2 was also identical to the procedure of Experiment 1, except that the participants assigned to the disease condition had to finish this experiment by using a very dirty keyboard, while the participants assigned to the control condition had to finish the experiment by using a normal keyboard. Participants in Experiment 2 did not have to finish the PVD scale.

### Results

**Fig 4:**
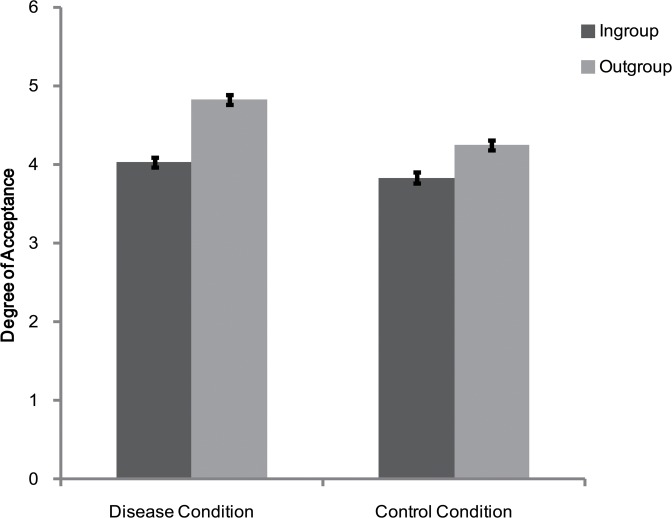
Degree of acceptance of faces labeled as ingroup members and outgroup members in Experiment 2. Error bars represent standard errors.

Rating scores for ingroup and outgroup members were subjected to a 2 (personality type: red, green) × 2 (category label: ingroup, outgroup) × 2 (environmental setting: disease condition, control condition) mixed-model ANOVA. The results showed that the main effect of category label [*F*(1, 76) = 78.33, *p* < 0.01, η_*p*_^2^ = 0.51], the main effect of environmental setting [*F*(1, 76) = 36.39, *p* < 0.01, η_*p*_^2^ = 0.32], and the interaction between these two variables [*F*(1, 76) = 7.31, *p* < 0.01, η_*p*_^2^ = 0.09] were all significant. Consistent with Experiment 1, participants preferred the outgroup members over the ingroup members under all environmental settings (disease condition: *F*(1, 38) = 55.46, *p* < 0.01, η_*p*_^2^ = 0.59; control condition: *F*(1, 38) = 23.71, *p* < 0.01, η_*p*_^2^ = 0.38; see Fig. 4). However, the main effect of personality type [*F*(1, 76) = 0.29, *p* > 0.05, η_*p*_^2^ = 0.004], the interaction between personality type and category label [*F*(1, 76) = 2.81, *p* > 0.05, η_*p*_^2^ = 0.04], and the interactions of all these three independent variables [*F*(1, 76) = 1.23, *p* > 0.05, η_*p*_^2^ = 0.02], were not significant.

To further illustrate the interaction between category label and environmental setting, rating scores of outgroup members in the face appraisal task were subtracted by that scores of ingroup members to create a composite score of ingroup derogation, and we subjected this score to a 2 (personality type: red, green) × 2 (environmental setting: disease condition, control condition) ANOVA. The results showed that the main effect of personality type [*F*(1, 76) = 2.81, *p* > 0.05, η_*p*_^2^ = 0.04] was not significant, but the main effect of environmental setting [*F*(1, 76) = 7.31, *p* < 0.01, η_*p*_^2^ = 0.09] was significant, with participants showing more ingroup derogation attitudes in the disease condition (*M* = 0.79, *SD* = 0.69) than in the control condition (*M* = 0.42, *SD* = 0.54). The interaction between personality type and environmental setting [*F*(1, 76) = 1.23, *p* > 0.05, η_*p*_^2^ = 0.02] was not significant. Therefore, the results of Experiment 2 indicated that the attitudes of ingroup derogation were exaggerated when there were cues of diseases in the immediate physical environment.

Additional analysis revealed the source of this "dirty keyboard" effect. Participants were more positive toward the outgroup members in the disease condition than in the control condition [*F*(1, 77) = 41.78, *p* < 0.01, η_*p*_^2^ = 0.35] (see Fig. 4), but the attitudes toward ingroup members were not affected by this manipulation [*F*(1, 77) = 3.71, *p* > 0.05, η_*p*_^2^ = 0.05].

## Experiment 3

In Experiment 3, we tested whether the Chinese participants would exaggerate their attitudes of ingroup derogation when the cues of diseases were from the immediate social environment (directly from the people) instead of the immediate physical environment. Specifically, we examined whether Chinese participants exaggerated their ingroup derogation attitudes even when both the ingroup and outgroup members were displaying the cues of diseases and whether the effects of disease cues were stronger for ingroup members. Considering that Experiment 1 and Experiment 2 showed that the assignment of personality type was irrelevant to the results, in Experiment 3, all participants were assigned as red personality type.

### Ethics Statement

The experimental procedures were approved by the IRB of the Institute of Psychology, Hunan Normal University. All participants provided written informed consent before taking part in the experiment and were debriefed after the experiment.

### Participants and Design

Thirty paid volunteers, all Chinese undergraduate or postgraduate students (15 males and 15 females), aged 19–27 years (mean age: 22.6 years, *SD* = 2.18), participated in this experiment. A 2 (category label: ingroup, outgroup) × 2 (health condition: control, disease) within-subjects design was used.

### Materials and Procedure

The bogus personality test and the facial stimuli employed by Experiment 3 were identical to those of Experiment 1, except that in Experiment 3 half of the facial stimuli of ingroup and outgroup members were randomly labeled with a pentacle to indicate that these people were infected with diseases and all participants were informed that they had a red personality.

The procedure was also identical to the procedure of Experiment 1 except that participants in Experiment 3 did not have to complete the PVD scale and before the face appraisal task they were informed that people labeled with a pentacle were infected with diseases.

### Results

**Fig 5:**
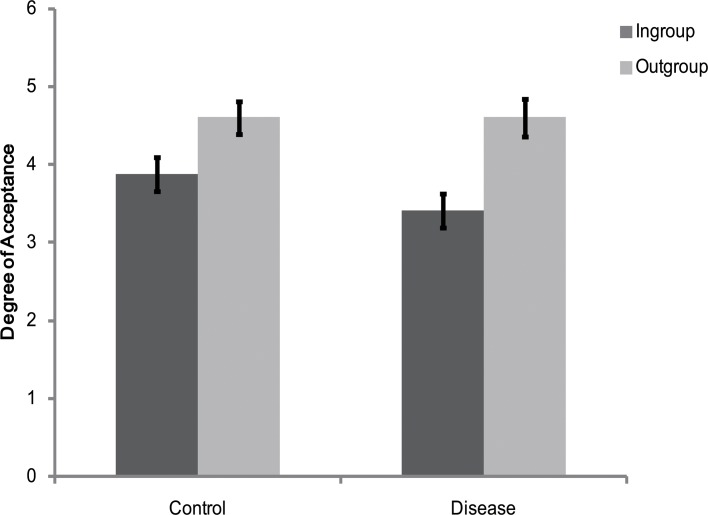
Degree of acceptance of faces labeled as ingroup members and outgroup members in Experiment 3. Error bars represent standard errors.

Rating scores for ingroup and outgroup members were subjected to a 2 (category label: ingroup, outgroup) × 2 (health condition: control, disease) repeated-measures ANOVA. The results showed that the main effect of category label [*F*(1, 29) = 13.99, *p* < 0.01, η_*p*_^2^ = 0.33] was significant. The main effect of health condition [*F*(1, 29) = 1.77, *p* > 0.05, η_*p*_^2^ = 0.06] was not significant. However, the interaction between category label and health condition [*F*(1, 29) = 9.59, *p* < 0.01, η_*p*_^2^ = 0.25] was significant. Simple effects analysis showed that the effect of disease cues [*F*(1, 29) = 6.31, *p* < 0.05, η_*p*_^2^ = 0.18] was significant for ingroup members but non-significant for outgroup members [*F*(1, 29) = 0.000, *p* > 0.05, η_*p*_^2^ = 0.000]. The participants were more likely to reject an ingroup member when it was infected with diseases, but no such effects were observed for outgroup members (see Fig. 5), which is consistent with our prediction. Simple effects analysis also showed that, participants consistently derogated ingroup members under all health conditions [control: *F*(1, 29) = 9.64, *p* < 0.01, η_*p*_^2^ = 0.25; disease: *F*(1, 29) = 16.17, *p* < 0.01, η_*p*_^2^ = 0.36] (see Fig. 5).

To further illustrate the interaction between category label and health condition, rating scores of outgroup members in the face appraisal task were subtracted by that scores of ingroup members to separately create a composite score of ingroup derogation for each health condition. A pairwise *t*-test showed that, as predicted, participants exaggerated their ingroup derogation attitudes even when ingroup and outgroup members were both infected (control: *M* = 0.73, *SD* = 1.29; disease: *M* = 1.19, *SD* = 1.63), *t*(29) = 3.1, *p* < 0.01.

## Discussion

Ingroup derogation is a strange anti-us tendency mainly reported among actual social groups [37–48]. To our knowledge, this is the first study investigating ingroup derogation toward normal ingroup members in artificially constructed groups. With three experiments, the present study investigated the phenomenon of ingroup derogation by using the minimal group paradigm. The groups created by minimal group paradigm were only artificial groups which had no real differences between each other. The group labels given to the group members were only heuristic cues and could only function to imply one's actual social group membership. There were no differences in group status and no group members were labeled as deviants. However, participants in the three experiments still consistently derogated their ingroup members. They were more likely to choose an outgroup member rather than an ingroup member to be their partner to work with.

The three experiments extended our previous study [44] by demonstrating that the mainland Chinese not only viewed the faces of outgroup members as more beautiful [44], but they were also more inclined to cooperate with outgroup members. These results were also consistent with previous studies [40–44, 49, 50–54]: For example, Chinese participants were reported to make outgroup-favoring and ingroup-disfavoring attributions [49], and they associated more negative attributes with their ingroup members than with their outgroup members [43]. In addition, these results showed that the mechanism of ingroup derogation responds to heuristic cues that only imply one's actual group membership, indicating this mechanism follows the smoke detector principle [1, 2, 36]. Therefore, these results support our hypothesis that ingroup derogation is related to the response of behavioral immune system.

Although relating the phenomenon of ingroup derogation to the threat management mechanism [70], the results as mentioned above can not specify which kind of threat the mechanism of ingroup derogation responds to. Studies have shown that ingroup favoritism can be triggered by internal and external cues of diseases. When individuals felt vulnerable to infectious diseases (internal cues), and when there were cues of diseases in the immediate physical environment (external cues) or from the immediate social environment (external cues), ingroup favoritism was reported to be exaggerated [10, 13, 20, 21, 25–34]. According to our hypothesis, if the mechanism of ingroup derogation was indeed a similar but complete opposite mechanism of ingroup favoritism—a special manifestation of the behavioral immune system that function to deal with a special situation in which ingroup members pose more threat of diseases than outgroup members—then the mechanism of ingroup derogation should follow the same functional flexibility principle and respond to the same cues of diseases as found in studies of ingroup favoritism, which means that the mechanism of ingroup derogation becomes more activated when individuals internally feel more vulnerable to diseases or become more vulnerable to diseases due to external causes.

Across three experiments, we have found the results that are consistent with the predictions we mentioned above. In Experiment 1, we measured individual differences in perceived vulnerability to diseases by using PVD scale [65]. The results showed that there were positive associations between the measures of perceived vulnerability to diseases and the degree of ingroup derogation. In Experiment 2, we manipulated the environment settings by instructing the participants to finish the experiment on a very dirty keyboard which was demonstrated to be an effective cue of diseases in a pilot study. The results showed that, compared with those in the control condition, participants in the disease condition exaggerated their attitudes of ingroup derogation. In Experiment 3, we tested the effects of disease cues when these cues externally originated from the immediate social environment rather than from the immediate physical environment. The results indicated that, the Chinese participants exaggerated their ingroup derogation attitudes even when ingroup and outgroup members were both displaying cues of diseases. Therefore, these results demonstrate that the activation of ingroup derogation mechanism is related to disease cues with internal or external origins. They also suggest that the mechanism of ingroup derogation is a threat management mechanism responding to the threat of diseases and it follows the functional flexibility principle.

According to our hypothesis, ingroup derogation is a special adaptation to a particular situation in which ingroup members pose more threat of diseases than outgroup members. To support such a hypothesis, the mechanism of ingroup derogation should not only follow the smoke detector and functional flexibility principles, but also should respond more consistently to ingroup members and respond more strongly to the disease threat incurred by ingroup members. In two of the three experiments, the results were consistent with this prediction. In Experiment 1, we found that PVD measures were negatively correlated with ingroup attitudes, but they were not associated with outgroup attitudes. In Experiment 3, we found that Chinese participants responded more strongly only when the ingroup members were infected (effects of disease cues were only significant for ingroup members). Furthermore, in Experiment 3, the attitudes of ingroup derogation were exaggerated even when both ingroup and outgroup members were displaying disease cues, which could happen only when the ingroup members posed more disease threat than outgroup members. These results suggest the mechanism of ingroup derogation is specialized to deal with the greater threat of diseases incurred by ingroup members. However, could this mechanism be so specialized that it disregards explicit disease-relevant information mediated by outgroup members as shown in Experiment 3? While the result of Experiment 1 suggests that the answer might be "yes" (i.e., we found the PVD scores were not correlated with outgroup attitudes), to a large extent, current results still leave the question unanswered. The methodology of the current study has limited the generalizability of our findings. In current study, participants had to face both the ingroup and outgroup members, making the differentiation between "us" and "them" being salient. Since interacting with ingroup members may bring greater costs and the cognitive resources of the individual are also limited, such a differentiation might lead the behavioral immune system to become so selectively responsive that it almost exclusively responds to ingroup members in order to achieve greatest efficiency for disease avoidance. One previous study has also found that participants show heightened attention to heuristic disease cues [9]. If the participants were facing the ingroup or outgroup members separately, the response pattern of behavioral immune system might be different.

Is ingroup derogation a result of ingroup avoidance or outgroup attraction? While the results of Experiment 1 and Experiment 3 suggest it is mainly driven by ingroup avoidance, the results of Experiment 2 suggest that, for ingroup derogation, the source of the disease threat matters. That is, the mechanism increased outgroup attraction but not ingroup avoidance when the disease cues were from the immediate physical environment. This response pattern was different from what was found in Experiment 1 and Experiment 3 in which the disease cues originated from people (internally or externally). However, this result is still compatible with our hypothesis: A disease threat from the physical environment means the physical environment itself is not safe; an attraction toward outsiders when facing an unsafe physical environment may help the individual to leave the local habitat in order to avoid the infection from the physical environment, and most importantly, it helps the individual to avoid dangerous multiple infection from ingroup members by leaving the local habitat, since physiological immune system will be compromised after the disease infection [9]; when the disease cues originated from the people (both internally and externally), the mechanism should respond to the person posing the disease threat, and in Experiment 1 and Experiment 3, these persons were mainly perceived to be the ingroup members. Similar effects can also be observed in the study of ingroup favoritism. For example, researchers found that when using Disgust Scale as a prime of disease, which contains many cues of disease threat from the physical environment rather than from a specific group member, the U.S. participants only increased their ingroup attraction but not the outgroup avoidance [26]. Therefore, the results of Experiment 2 suggest ingroup derogation can also be driven by outgroup attraction. Of course, more rigorous tests and experiments are needed before clear conclusions can be drawn about the root of ingroup derogation and the effects of source of disease threats.

In summary, the current results support our hypothesis. These results suggest that the mechanism of ingroup derogation is related to the evolved response of behavioral immune system, and it is specialized to deal with a special ecological condition in which greater threat of diseases is incurred by ingroup members. Although the current evidence from the three experiments is consistent with our predictions, it is still necessary to be cautious and consider the evidence as preliminary. Direct tests of such an evolutionary hypothesis concerning human social lives are quite difficult, since it is impossible for researchers to have a time machine or to observe any clear fossil records about social interactions. However, more direct tests (e.g., direct manipulation of relative pathogen load between social groups, which seems to be difficult either; or to conduct behavioral genetic research) and cross culture studies have to be employed to come up with a more clear view of the function of ingroup derogation mechanism.

The hypothesis we proposed in present study can explain not only the phenomenon of ingroup derogation found among mainland Chinese participants, but also the ingroup derogation found against deviant ingroup members (black sheep effect) [45–48]. Numerous studies on behavioral immune system have shown that this system is designed to facilitate the avoidance of the individuals who seem to be deviant from the "normal" (e.g. obesity, physical disabilities, elders) [16, 17, 21–23], which is a manifestation of the smoke detector principle. The same logic can be applied to deviant ingroup members: Deviant from social norms is a heuristic cue of diseases, interaction with these individuals could be dangerous, thus derogation and avoidance toward such individuals would offer some evolutionary advantages [14, 15, 26, 66]. Researchers should explore the relationship between the threat of diseases and the black sheep effect in the future.

It should be noted that current results cannot explain the ingroup derogation found in minority groups with an inferior social status [37–39]: An inferior minority group identity cannot be related to diseases, and the group status of the two artificial groups created by the current study was also equal. Such an intergroup bias found in the minority groups is more possibly related to the resource competition or the threat of physical violence [35, 36] than to the threat of diseases. That is, with an inferior social status, resources distributed to a specific group become quite limited, thus exacerbating ingroup resource competition. Such a situation might lead the interactions with ingroup members to become more costly and dangerous than those with outgroup members. Under such circumstances, it would be more adaptive to affiliate with outgroup members than with ingroup members. This possibility demands investigation in the future.

It also needs to be noted that, while the current results suggest a potential causal link between pathogen threat and ingroup derogation, the exact mechanism that accounts for that link has not been identified. A variety of different mechanisms are plausible. For example, ingroup derogation may be shaped by differential genetic selection, or by the differential developmental expression of common genes. The most plausible mechanism, however, may be that ingroup derogation is caused by the differential activation of functional flexible neurocognitive mechanisms: The selection pressure of pathogen threat has shaped the psychological mechanisms of intergroup attitudes as a whole, leading individuals to harbor ingroup favoritism when it is safer to stay with ingroup members but to endorse ingroup derogation attitudes while it is not. Careful elucidation of these underlying mechanisms may help us to explain the phenomenon that we observe ingroup derogation in some cultures (e.g. China) but finding the reverse effect (ingroup favoritism) in other contexts (e.g., different cultures may have different ecological conditions, which cause differential activation of a same neurocognitive mechanism). This is an important direction for future research.

In the current study, participants showed ingroup derogation attitudes only in the domain of cooperation. If the mechanism of ingroup derogation is indeed evolutionarily rooted, its activation would result in other functionally related changes, such as enhanced attention, perception, and memories to threat-specific targets [70, 71]. To ensure the phenomenon has its root in human evolution, studies are also needed to investigate the other cognitive domains as mentioned above.

## Supporting Information

S1 Raw Data Raw data files of the current study. (ZIP)

## References

[1] Schaller M, Park JH. The behavioral immune system (and why it matters). Curr Dir Psychol Sci. 2011; 20: 99–103.

[2] Schaller M. The behavioural immune system and the psychology of human sociality. Phil Trans R Soc B. 2011; 366: 3418–3426. 10.1098/rstb.2011.0029 22042918 PMC3189350

[3] Hart BL. Behavioral adaptations to pathogens and parasites: Five strategies. Neurosci Biobehav Rev. 1990; 14: 273–294. 2234607 10.1016/s0149-7634(05)80038-7

[4] Kavalier M, Choleris E, Pfaff DW. Recognition and avoidance of the odors of parasitized conspecifics and predators: differential genomics correlates. Neurosci Biobehav Rev. 2005; 29: 1347–1359. 16055189 10.1016/j.neubiorev.2005.04.011

[5] Kiesecker JM, Skelly DK, Beard KH, Preisser E. Behavioral reduction of infection risk. Proc Nat Acad Sci USA. 1999; 96: 9165–9168. 10430913 10.1073/pnas.96.16.9165 PMC17750

[6] Mortensen CR, Becker DV, Ackerman JM, Neuberg SL, Kenrick DT. Infection breeds reticence: the effects of disease salience on self-perceptions of personality and behavioral avoidance tendencies. Psychol Sci. 2010; 21: 440–447. 10.1177/0956797610361706 20424082

[7] Schaller M, Murray DR. Pathogens, personality and culture: disease prevalence predicts worldwide variability in sociosexuality, extraversion, and openness to experience. J Pers Soc Psychol. 2008; 95: 212–221. 10.1037/0022-3514.95.1.212 18605861

[8] Curtis V, de Barra M, Aunger R. Disgust as an adaptive system for disease avoidance behavior. Phil Trans R Soc B. 2011; 366: 389–401. 10.1098/rstb.2010.0117 21199843 PMC3013466

[9] Miller SL, Maner JK. Sick body, vigilant mind: The biological immune system activates the behavioral immune system. Psychol Sci. 2011; 22: 1467–1471. 10.1177/0956797611420166 22058109

[10] Miller SL, Maner JK. Overperceiving disease cues: the basic cognition of the behavioral immune system. J Pers Soc Psychol. 2012; 102: 1198–1213. 10.1037/a0027198 22329656

[11] Tybur JM, Bryan AD, Magnan RE, Hooper AEC. Smells like safe sex: olfactory pathogen primes increase intentions to use condoms. Psychol Sci. 2011; 22: 478–480. 10.1177/0956797611400096 21350181

[12] Murray DR, Jones DN, Schaller M. Perceived threat of infectious disease and its implications for sexual attitudes. Pers Individ Dif. 2013; 54: 103–108.

[13] Fincher CL, Thornhill R, Murray DR, Schaller M. Pathogen prevalence predicts human cross-cultural variablity in individualism/collectivism. Proc R Soc B. 2008; 275: 1279–1285. 10.1098/rspb.2008.0094 18302996 PMC2602680

[14] Murray DR, Schaller M. Threats and conformity deconstructed: perceived threat of infectious disease and its implications for conformist attitudes and behavior. Eur J Soc Psychol. 2012; 42: 180–188.

[15] Murray DR, Trudeau R, Schaller M. On the origins of cultural differences in conformity: four tests of the pathogen prevalence hypothesis. Pers Soc Psychol Bull. 2011; 37: 318–329. 10.1177/0146167210394451 21307175

[16] Ryan S, Oaten M, Stevenson RJ, Case TI. Facial disfigurement is treated like an infectious disease. Evol Hum Behav. 2012; 33: 639–646.

[17] Park JH, Leeuwen FV, Chochorelou Y. Disease-avoidance processes and stigmatization: cues of substandard health arouse heightened discomfort with physical contact. J Soc Psychol. 2013; 153: 212–228. 23484348 10.1080/00224545.2012.721812

[18] John T, Natalie JS, Michael AM. The behavioral immune system and social conservatism: a meta-analysis. Evol Hum Behav. 2013; 34: 99–108.

[19] Cashdan E, Steele M. Pathogen prevalence, group bias, and collectivism in the standard cross-cultural sample. Hum Nat. 2013; 24: 59–75. 10.1007/s12110-012-9159-3 23389437

[20] Huang JY, Sedlovskaya A, Ackerman JM, Bargh JA. Immunizing against prejudice: effects of disease protection on attitudes toward out-groups. Psychol Sci. 2011; 22: 1550–1556. 10.1177/0956797611417261 22058107

[21] Lund EM, Miller SL. Is obesity un-American? disease concerns bias implicit perceptions of national identity. Evol Hum Behav. 2014; 35: 336–340.

[22] Park JH, Faulkner J, Schaller M. Evolved disease-avoidance processes and contemporary anti-social behavior: prejudicial attitudes and avoidance of people with physical disabilities. J Nonverbal Behav. 2003; 27: 65–87.

[23] Park JH, Schaller M, Crandall CS. Pathogen-avoidance mechanisms and the stigmatization of obese people. Evol Hum Behav. 2007; 28: 410–414.

[24] Cottrell CA, Neuberg SL. Different emotional reactions to different groups: a sociofunctional threat-based approach to "prejudice". J Soc Psychol. 2005; 88: 770–789. 15898874 10.1037/0022-3514.88.5.770

[25] Navarrete CD, Fessler DMT, Eng ST. Elevated ethnocentrism in the first trimester of pregnancy. Evol Hum Behav. 2007; 28: 60–65.

[26] Navarrete CD, Fessler DMT. Disease, avoidance and ethnocentrism: the effects of disease vulnerability and disgust sensitivity on intergroup attitudes. Evol Hum Behav. 2006; 27: 270–282.

[27] Faulkner J, Schaller M, Park JH, Duncan LA. Evolved disease-avoidance mechanisms and contemporary xenophobic attitudes. Group Process Intergroup Relat. 2004; 7: 333–353.

[28] Letendre K, Fincher CL, Thornhill R. Does infectious disease cause global variation in frequency of intrastate armed conflict and civil war. Biol Rev Camb Philos Soc. 2010; 85: 669–683. 10.1111/j.1469-185X.2010.00133.x 20377573

[29] Thornhill R, Fincher CL, Aran D. Parasites, democratization, and the liberalization of values across contemporary countries. Biol Rev Camb Philos Soc. 2009; 84: 113–131. 10.1111/j.1469-185X.2008.00062.x 19046399

[30] Leeuwen F, Park JH, Koenig BL, Graham J. Regional variation in the pathogen prevalence predicts endorsement of group-focused moral concerns. Evol Hum Behav. 2012; 33: 429–437.

[31] Fincher CL, Thornhill R. Parasite-stress promotes in-group assortative sociality: the case of strong family ties and heightened religiosity. Behav Brain Sci. 2012; 35: 61–79. 10.1017/S0140525X11000021 22289223

[32] Schaller M, Murray DR. Infectious disease and the evolution of cross-cultural difference In: Schaller M, editor. Evolution, culture, and the human Mind. New York: Psychology Press; 2010 pp. 243–256.

[33] Fincher C, Thornhill R. Assortative sociality, limited dispersal, infectious disease and the genesis of the global pattern of religion diversity. Proc R Soc B. 2008; 275: 2587–2594. 10.1098/rspb.2008.0688 18664438 PMC2605802

[34] Fincher C, Thornhill R. A parasite-driven wedge: infectious diseases may explain language and other biodiversity. Oikos. 2008; 117: 1289–1297.

[35] Van Vugt M, Park JH. Guns, germs, and sex: how evolution shaped our intergroup psychology. Soc Personal Psychol Compass. 2009; 3: 927–938.

[36] Schaller M, Neuberg SL. Danger, disease, and the nature of prejudice(s) In Olson JM, Zanna MP, editors. Advances in experimental social psychology, Vol. 46 Burlington: Academic Press; 2012 pp. 1–54.

[37] Allport G. The nature of prejudice. Cambridge, MA: Addison Wesley; 1958 (Original work published 1954).

[38] Jost JT, Pelham BW, Carvallo M. Non-conscious forms of system justication: cognitive, affective, and behavioral preferences for high status groups. J Exp Soc Psychol. 2002; 38: 586–602.

[39] Jost JT. Outgroup favoritism and the theory of system justification: an experimental paradigm for investigating the effects of socio-economic success on stereotype content In Moskowitz G, editor. Cognitive social psychology: the Princeton symposium on the legacy and the future of social cognition. Mahwah, NJ: Erlbaum Hungarian Translation; 2001 pp. 89–102.

[40] Cuddy AJ, Fiske ST, Kwan VS, Glick P, Demoulin S, Leyens JP, et al Stereotype content model across cultures: towards universal similarities and some differences. Br J Soc Psychol. 2009; 48: 1–33. 10.1348/014466608X314935 19178758 PMC3912751

[41] Heine SJ, Lehman DR. The cultural construction of self-enhancement: an examination of group-serving biases. J Pers Soc Psychol. 1997; 72: 1268–1283. 9177019 10.1037//0022-3514.72.6.1268

[42] Snibbe AC, Kitayama S, Markus HR, Suzuki T. They saw a game: a Japanese and American (football) field study. J Cross Cult Psychol. 2003; 34: 581–595.

[43] Ma-Kellams C, Spencer-Rodgers J, Peng K. I am against us? unpacking cultural differences in ingroup favoritism via dialecticism. Pers Soc Psychol B. 2011; 37: 15–27. 10.1177/0146167210388193 21084525

[44] Zhao K, Wu Q, Shen XB, Xuan YM, Fu XL. I undervalue you but I need you: the dissociation of attitude and memory toward in-group members. PLoS ONE. 2012; 7: e32932 10.1371/journal.pone.0032932 22412955 PMC3296752

[45] Lewis AC, Sherman SJ. Perceived entitativity and the black-sheep effect: when will we denigrate negative ingroup members?. J Soc Psychol. 2010; 150: 211–225. 10.1080/00224540903366388 20397595

[46] Marques JM, Yzerbyt VY, Leyens JP (1988) The ‘Black Sheep Effect’: extremity of judgments towards ingroup members as function of group identification. Eur J Soc Psychol. 1988; 18: 1–16.

[47] Pinto IR, Marques JM, Levine JM, Abrams D. Membership status and subjective group dynamics: who triggers the black sheep effect?. J Pers Soc Psychol. 2010; 99: 107–119. 10.1037/a0018187 20565188

[48] Reese G, Steffens MC, Jonas KJ. When black sheep make us think: information processing and devaluation of in- and outgroup norm deviants. Soc Cogn. 2013; 31: 482–503.

[49] Hewstone M, Ward C. Ethnocentrism and causal attribution in Southeast Asia. J Pers Soc Psychol. 1985; 48: 614–623.

[50] Jahoda G, Thomson SS, Bhatt S. Ethnic identity and preferences among Asian immigrant children in Glasgow: a replicated study. Eur J Soc Psychol. 1972; 2: 19–32.

[51] Lee YT, Ottati V. Perceived in-group homogeneity as a function of group salience and stereotype threat. Pers Soc Psychol Bull. 1995; 21: 610–619.

[52] Lee YT, Ottati V. Determinants of in-group and outgroup perceptions of heterogeneity. J Cross Cult Psychol. 1993; 24: 298–318.

[53] Diener E, Suh EM., Smith H, Shao L. National differences in reported subjective well-being: why do they occur?. Soc Indic Res. 1995; 34: 7–32.

[54] Endo Y, Heine SJ, Lehman DR. Culture and positive illusions in close relationships: how my relationships are better than yours. Pers Soc Psychol Bull. 2000; 26: 1571–1586.

[55] Hruschka DJ, Henrich J. Institutions, parasites and the persistence of ingroup preferences. PLoS ONE. 2013; 8: e63642 10.1371/journal.pone.0063642 23704926 PMC3660589

[56] Talhelm T, Zhang X, Oishi S, Shimin C, Duan D, Lan X, et al Large-scale psychological differences within China explained by rice versus wheat agriculture. Science. 2014; 344: 603–608. 10.1126/science.1246850 24812395

[57] Fincher CL, Thornhill R. Parasite-stress theory may be a general theory of culture and sociality. Behav Brain Sci. 2012; 35: 99–119. 22486004 10.1017/s0140525x11001774

[58] Thornhill R, Fincher CL. The parasite-stress theory of values and sociality: infectious disease, history and human values worldwide. New York, NY: Springer; 2014.

[59] Bernstein MJ, Young SG, Hugenberg K. The cross-category effect. Psychol Sci. 2007; 18: 706–712. 17680942 10.1111/j.1467-9280.2007.01964.x

[60] Tajfel H, Billig M, Bundy R, Flament C. Social categorization and intergroup behaviour. Eur J Soc Psychol. 1971; 1: 149–178. 5100005

[61] Ruffle BJ, Sosis R. Cooperation and the in-group-out-group bias: a field test on Israeli kibbutz members and city residents. J Econ Behav Organ. 2006; 60: 147–163.

[62] Yamagishi T, Mifune N. Social exchange and solidarity: in-group love or out-group hate?. Evol Hum Behav. 2009; 30: 229–237.

[63] Rand DG, Pfeiffer T, Dreber A, Sheketoff RW, Wernerfelt NC, Benkler Y. Dynamic remodeling of in-group bias during the 2008 presidential elections. PNAS. 2009; 106: 6187–6191. 10.1073/pnas.0811552106 19332775 PMC2664153

[64] Eysenck HJ, Eyesenck SBG. Manual of the Eyesenck Personality Questionaire (Junior and Adult). Kent, UK: Hodder & Stoughton; 1975.

[65] Duncan LA, Schaller M, Park JH. Perceived vulnerability to disease: development and validation of a 15-item self-report instrument. Pers Individ Dif. 2009; 47: 541–546.

[66] Wu BP, Chang L. The social impact of pathogen threat: how disease salience influences conformity. Pers Individ Dif. 2012; 53: 50–54.

[67] Wong JJ, Cho SY. A local experts organization model with application to face emotion recognition. Expert Syst Appl. 2009; 36: 804–819.

[68] Luo W, Feng W, He W, Wang NY, Luo YJ. Three stages of facial expression processing: ERP study with rapid serial visual presentation. Neuroimage. 2010; 49: 1857–1867. 10.1016/j.neuroimage.2009.09.018 19770052 PMC3794431

[69] Wang S, Liu Z, Lv S, Lv Y, Wu G, Peng P, et al A natural visible and infrared facial expression database for expression recognition and emotion inference. IEEE T Multimedia. 2010; 12: 682–691.

[70] Neuberg SL, Kenrick DT, Schaller M. Human threat management systems: self-protection and disease avoidance. Neurosci Biobehav Rev. 2011; 35: 1042–1051. 10.1016/j.neubiorev.2010.08.011 20833199 PMC3024471

[71] Cottrell CA, Park JH. Evolutionary perspectives on prejudice In: Stangor C, Crandall C, editors. Stereotyping and prejudice. New York: Psychology Press; 2013 pp. 29–52.

